# A bolder One Health: expanding the moral circle to optimize health for all

**DOI:** 10.1186/s42522-021-00053-8

**Published:** 2021-12-07

**Authors:** Simon Coghlan, Benjamin John Coghlan, Anthony Capon, Peter Singer

**Affiliations:** 1 University of Melbourne, Melbourne, Australia; 2 Burnet Institute, Melbourne, Australia; 3 Monash Sustainable Development Institute, Monash University, Melbourne, Australia; 4 Princeton University, Princeton, NJ USA

**Keywords:** One Health, Ethics, Anthropocentrism, Philosophy, Health determinants, Radical change

## Abstract

One Health is a ground-breaking philosophy for improving health. It imaginatively challenges centuries-old assumptions about wellbeing and is now widely regarded as the ‘best solution’ for mitigating human health problems, including pandemic zoonotic diseases. One Health’s success is imperative because without big changes to the status quo, great suffering and ill-health will follow. However, even in its more ambitious guises, One Health is not radical enough. For example, it has not embraced the emerging philosophical view that historical anthropocentrism is an unfounded ethical prejudice against other animals. This paper argues that One Health should be more imaginative and adventurous in its core philosophy and ultimately in its recommendations and activities. It must expand the circle of moral concern beyond a narrow focus on human interests to include nonhuman beings and the environment. On this bolder agenda, progressive ethical and practical thinking converge for the benefit of the planet and its diverse inhabitants—human and nonhuman.

## Introduction

One Health is a ground-breaking philosophy for improving health. It imaginatively challenges centuries-old assumptions about wellbeing and is now widely regarded as the ‘best solution’ for mitigating human health problems, including pandemic zoonotic diseases [1]. One Health’s success is imperative because without big changes to the status quo, great suffering and ill-health will follow. However, even in its more ambitious guises, One Health is not radical enough. For example, it has not embraced the emerging philosophical view that historical anthropocentrism is an unfounded ethical prejudice against other animals. This paper argues that One Health should be more imaginative and adventurous in its core philosophy and ultimately in its recommendations and activities. It must expand the circle of moral concern beyond a narrow focus on human interests to include nonhuman beings and the environment. On this bolder agenda, progressive ethical and practical thinking converge for the benefit of the planet and its diverse inhabitants—human and nonhuman.

## The existing One Health philosophy

Before the 21^st^ Century revival in One Health-style thinking, human health problems were tackled largely downstream from their root causes [2]. Even when broader health determinants were considered, these were mostly constructed in social and economic terms without consideration of other species and the wider environment [3, 4]. One Health broke ground by refocusing modern scientific attention and practical action on the upstream influences on health. It recognised and described the close interconnectedness of human, animal, and environmental health and thus the need for intimate collaboration between medical, veterinary, and ecological sciences. This radical approach can generate “adaptive, forward-looking and multidisciplinary solutions” [5] to attain, as the seminal One Health Initiative Final Report says, “optimal health for people, animals, and our environment” [6]. But despite this transformational thinking, the core philosophy of One Health needs to be much more progressive. There are two linked components of this bolder philosophy: one ethical, the other practical.

## An ethically bolder One Health

Human understanding can progress over time across various domains of thought. This is clear in the case of science, which has deepened our understanding of the natural world. Philosophy too can progress, including in ethics [7]. While a deepened ethical understanding is partly driven by social and cultural changes, philosophers, particularly since the 1970s, have articulated strong arguments against our legacy of extreme anthropocentrism [8]. According to such anthropocentrism, only human beings have significant intrinsic moral value or worth, and humans alone deserve strong ethical protection. In contrast, nonhuman beings and entities warrant no or minimal moral consideration and may, within wide limits, be routinely harmed for human ends.

Many moral thinkers now oppose such anthropocentrism. On one view, advancing moral understanding historically involves an ‘expanding circle’ of moral concern that reaches beyond family and clan to include other individuals and groups whose wellbeing does not directly affect us [7]. An expanding circle of moral concern means, first of all, that we have strong duties to *all* humans, including peoples whose appearance and culture differ from our own. The vital interests of distant and different peoples deserve greater moral consideration irrespective of any benefits that people in more fortunate circumstances might receive from extending assistance to them.

This expanding circle also means granting greater moral consideration to nonhuman animals. Although contemporary philosophers espouse differing theoretical perspectives on morality—including utilitarianism, deontology, feminist ethics, and virtue theory—many of these philosophers nonetheless agree that human societies have systematically and wrongly disregarded the interests of sentient nonhuman animals. For example, wild animals are often unjustly killed in land-clearing operations and deliberately culled with next to no moral consideration. Perhaps most grotesquely, billions of sentient domestic animals are wrongfully made to suffer pain, distress, and deprivation their entire lives in industrial factory farms [8].

Recognising a much higher moral status in nonhuman animals means seriously weighing their interests in moral deliberation. Some thinkers believe that comparable nonhuman and human interests deserve *equal* consideration; others that human interests weigh more heavily but that nonhuman interests nonetheless must be taken seriously. For most philosophers, morally relevant interests certainly include those of *sentient* creatures, such as mammals and birds, because sentient creatures can suffer and be miserable or happy [8].

Scientists and philosophers have recently argued that other vertebrates and some invertebrates also probably have sentience. This emerging view is important given that human activity, not least industrial fishing, harms trillions of such animals each year [9]. Some philosophers believe that certain cognitive and emotional capacities generate more complex interests worthy of moral concern. This might include animals that have sophisticated preferences to belong to a group, to engage in particular rewarding activities, and to avoid captivity or being killed [10].

The moral circle may be expanded still further. Bio-centrist and eco-centrist thinkers argue that non-sentient living things, ecosystems, and natural collectives like species have intrinsic moral value [11]. This view that *non*-sentient entities have intrinsic moral status is important, though it remains philosophically controversial. However, even thinkers who deny intrinsic moral status to non-sentient entities nonetheless often argue that our duties to protect the natural world for the sake of sentient beings are much stronger than our anthropocentric societies have acknowledged.

To a limited degree, One Health already gestures towards a widened moral circle [12]. However, the health and wellbeing of the nonhuman world continues to be effectively regarded as merely instrumental to narrowly construed human ends. Our argument is that One Health should reform itself to incorporate ethical and philosophical advances in thinking and adopt the idea of a truly expanded moral circle into its core philosophy.

## A practically bolder One Health

The above ethical imperatives have broad and fundamental practical implications. A progressive One Health that rejects historical anthropocentric prejudice will not simply accept and work within systems and practices that drive health problems, but will instead question those entrenched and ethically unjustified practices that often form part of the unseen background. This requirement follows from full expansion of the moral circle. Such expansion can amplify the positive impact of One Health on peoples in low-income countries, nonhuman animals, and the environment, while also significantly converging with much of what is strategically required to foster our own wellbeing [13]. The following considerations and steps are especially important.

Recent diseases that have spread from animals like Ebola and Zika have disproportionately affected the poor, and zoonotic diseases in general are most likely to arise in some of the world’s poorest places. Yet there has been inadequate global implementation of the prime instrument to strengthen global health security—the International Health Regulations 2005—and a chronic underinvestment in health in many parts of the world. Health security and universal health coverage are only just beginning to be linked [14]. The Planetary Health Manifesto published by *The Lancet* in 2014 calls for scientists to “minimize differences in health according to wealth, education, gender and place” [15]. Addressing global human health inequity should be a One Health priority.

One of the greatest but often neglected causes of global suffering and ill-health is industrial animal agriculture. Although intensive animal farming produces benefits, these are mostly morally outweighed by harms to animals given that there are alternative means of feeding ourselves. Conventional One Health works within the parameters set by intensive farming, such as by enhancing biosecurity and monitoring pathogens. By contrast, a bolder One Health philosophy will recognize that much animal agriculture unjustifiably imposes terrible suffering on billions of sentient animals, as well contributing to biodiversity loss and catastrophic climate change [16]. Incredibly, the global biomass of livestock now far exceeds that of all wild mammals [17].

Mass conversion of land used for animal production to native vegetation, cropping, and biofuel production would simultaneously improve human, animal, and environmental health [18]. Rewilding huge amounts of land cleared for animal farming and for inefficiently producing crops to feed those animals should be a core philosophical position. One Health also ought to recognise the need for massively expanding plant-based diets (and perhaps boosting investment in lab-based meat alternatives) to encourage a profound shift away from animal meat production. Plant-based food alternatives generally avoid wronging animals, are better for human health, mitigate antimicrobial resistance, and are vital for tackling global warming.

Intensive farming has played a role in previous deadly zoonotic disease outbreaks, including avian influenza viruses arising in Asia and the 2009 H1N1 influenza virus from North American piggeries. In fact, domestic mammals are among the most central species for sharing of zoonotic viruses with humans [19]. To some extent, the wildlife trade and some kinds of wet markets (live-animal and wildlife markets) treat wild animals unethically and raise risks of pathogens leaping from animals to humans [20]. These practices should in general be carefully phased out in all countries [21], whilst vigorously addressing the black-market trade and creating opportunities for new livelihoods. Although it is important to better understand the pathways of disease emergence for tailored preventive efforts, One Health’s core philosophy should not find it sufficient to research and monitor pathogens without also challenging practices that are already known to drive the risk of disease emergence.

One Health can support better global education about our ethical responsibilities for the planet and its inhabitants. Morally inclusive lessons about the interests and mutual interdependency of people, animals, and the natural world need to be taught at all levels, including but not limited to disciplines like medicine and veterinary science that influence One Health practice [22]. A less human-centered One Health philosophy could be a powerful vehicle for educating people about the ethical and practical changes that are required to save and protect our planet [23].

None of this means that there will not be difficult moral trade-offs between humans, animals, and the environment. For example, addressing climate change and damaging farming practices may have negative consequences for certain individuals, not least poor people. Consider, for example, how polices that unintentionally cause people to switch from hunting wildlife to domestic animal production could be counterproductive. However, expanding the moral circle is consistent with recognising the need for hard and just ethical thinking about the distribution of harms and benefits amongst humans and nonhumans.

## Conclusion

The One Health philosophy has simultaneously been ground-breaking and too conventional. On the one hand, it offers a radical program of reimagining human and nonhuman co-vulnerability and its effects on health. On the other hand, it has not yet embraced the progress made in philosophy and science that has exposed historical anthropocentrism as an entrenched prejudice. While it is important to pursue specific and small-scale research activities and programs, deeply appreciating the bigger moral and strategic picture as part of One Health’s central philosophy is urgent and vital. To some extent, exactly how One Health activities and policies should be changed by these core ethical and practical transformations is a matter for further debate. But a One Health that expands the moral circle and boldly reimagines our relations with other humans, nonhuman animals, and the environment is required if the movement is to achieve its aim of optimizing the health of the planet and its many different inhabitants.

## Data Availability

Not applicable.
